# Supplementary material for: Compressed Brown Algae as a Potential Environmental Enrichment Material in Growing Pigs
Source: Animals (Basel). 2021 Jan 27;11(2):315. doi: 10.3390/ani11020315 (PMC7912665; doi:10.3390/ani11020315)
Supplement: Supplementary file 1 [file animals-11-00315-s001.pdf]

Supplementary Table 1. Algae material composition given by the manufacturer (Algopack, Saint-Malo, France). The material is made with Saccharina latissimi, a brown macroalgae, post-harvest dried and compressed.

Average chemical composition
Dry matter (on fresh weight): 10 - 30 %.
Mineral matter (on dry matter): 14 - 34%.
Carbohydrates (on dry matter): 45 - 65%.
Proteins (on dry matter): 5 - 13%.
Lipids (on dry matter): 0.5 - 2%.
Organic matter (average in % of dry matter)
Alginic acid: 25 to 32
Laminarine: 4 to 14
Mannitol: 8 to 13
Cellulosic material: 7.5 to 8
Mineral materials (average in % of dry matter)
Sodium: 3.9 to 5
Potassium: 2 to 3.5
Nitrogen: 2.2
Sulfur: 1.5 to 3
Calcium: 1 to 2
Magnesium: 1
Phosphorus: 0.35
Trace elements (average in mg/kg of dry matter)
Iodine: 700 to 5000
Iron: 45 to 600
Zinc: 38 to 108
Nickel: 3 to 7
Copper: 5 to 6
Manganese: 1
Cobalt: 2
